# Case study of the Beijing 2022 Olympic Winter Games: Implications for global mass gathering events amidst the COVID-19 pandemic

**DOI:** 10.3389/fpubh.2023.1068023

**Published:** 2023-02-06

**Authors:** Da Huo, Ying Shen, Tao Zhou, Tong Yu, Ruoran Lyu, Ying Tong, Ting Gao, Quanyi Wang

**Affiliations:** ^1^Beijing Center for Disease Prevention and Control, Beijing, China; ^2^Beijing Organizing Committee for the 2022 Olympic and Paralympic Winter Games, Beijing, China; ^3^School of Public Health, Capital Medical University, Beijing, China; ^4^Beijing Municipal Key Laboratory of Clinical Epidemiology, Beijing, China; ^5^Beijing Economic-Technological Development Area Center for Disease Prevention and Control, Beijing, China

**Keywords:** COVID-19, Beijing 2022 Olympic Winter Games, bubble strategy, mass gathering events, N95 masks

## Abstract

**Objective:**

This study aimed to evaluate the public health countermeasures against coronavirus disease 2019 (COVID-19) that are important for organizing mass gathering events (MGEs) during a pandemic and to identify the practices suitable for application at future MGEs.

**Methods:**

This study analyzed data from the Beijing 2022 Olympic Winter Games. The aforementioned analysis was conducted from the viewpoints of overseas stakeholders and Chinese residents. The comprehensive set of countermeasures established to prevent the transmission of the COVID-19 pandemic comprised the bubble strategy, the three-layer testing strategy (pre-departure testing, testing at the airport, and daily screening), the mandatory wearing of N95 masks, and mandatory vaccination.

**Findings:**

A total of 437 positive cases within the bubble were reported during the Games, of which 60.6% were detected through screening at the airport and 39.4% were detected through routine screening. Nearly, 92.0% of the positive cases were detected within 7 days of arrival in China, and 80.8% of the cases had already been identified before the Opening Ceremony of the Games. Outside the bubble, no Games stakeholders were infected and no spectator contracted COVID-19. The bubble strategy, the three-layer testing strategy, the mandatory wearing of N95 masks, and mandatory vaccination are promising countermeasures to prevent the transmission of severe acute respiratory syndrome coronavirus 2 (SARS-CoV-2) during MGEs.

**Conclusion:**

Public health countermeasures introduced during the Beijing 2022 Olympic Winter Games were proven to be useful. The success in delivering and organizing the Games instills confidence and leaves a public health legacy for future MGEs amid the pandemic of COVID-19 or future emerging infectious diseases.

## Introduction

Since the emergence of coronavirus disease 2019 (COVID-19), numerous international travel regulations have been implemented to reduce the risk of the global spread of the pandemic. However, no consensus has been reached on the optimal public health measures for international MGEs. Emerging variants of concern have led to waves of global infection (1). Furthermore, constant severe acute respiratory syndrome coronavirus 2 (SARS-CoV-2) mutations along with uneven vaccine rollouts, hesitation in getting vaccinated, waning immunity, breakthrough infections, and the reluctance to accept nonpharmaceutical interventions made it highly possible for a number of countries to never achieve herd immunity (2). Under such circumstances, different countries embraced various strategies such as the “dynamic zero” and “living with the virus.” Regardless of the implemented policy, MGEs present a great challenge that could increase the risk of disease transmission intra- and internationally (3, 4).

The COVID-19 pandemic presents a considerable number of challenges in the political sphere, social activity, and public health, and many governments and organizations considered the cancellation or suspension of MGEs. Consequently, the Euro 2020 and Copa America, Tokyo 2020 Olympic Games, and Lucerne 2021 Winter Universiade were either postponed, canceled, or held without spectators to mitigate the risk of disease transmission (5).

The impact of the COVID-19 pandemic on large-scale sporting events is unprecedented, as previous international games have proceeded, despite the World Health Organization (WHO) declaring Public Health Emergency of International Concern (PHEIC). For example, the Vancouver 2010 Winter Olympic Games were held during the H1N1 influenza pandemic, the 2015 Africa Cup of Nations Football Tournament was held during the Ebola virus epidemic, and the Rio 2016 Olympic Games were held during the Zika virus outbreak (3, 6–8).

Although MGEs for sporting, religious, political, and other reasons potentially magnify the risk of infection, they are essential parts of social life that bring a sense of confidence and joy and are thus worth resuming as soon as possible (8–10). Unfortunately, outbreaks occurred almost every time an MGE was held during the COVID-19 pandemic. For instance, the national election was the major driver of two local outbreaks of COVID-19 in Malaysia during the second and third global waves (11). However, the Beijing 2022 Olympic Winter Games (hereafter referred to as the Beijing 2022 Games) were an exception, with all COVID-19 cases detected on time and no other major infectious diseases reported. The Beijing 2022 Games were held amid the very peak of the fourth COVID-19 wave when the more transmissible Omicron variant was prevalent (1). Although China embraced a different COVID-19 strategy than most other countries, its achievements and experiences in hosting such a large-scale international sporting event could provide some insights into public health preparedness for global MGEs amid future pandemics of emerging infectious diseases. The present study explored the rationale and success of epidemic control measures implemented in the Beijing 2022 Games to aid in public health preparedness for future MGEs during a pandemic.

## Methods

This case study evaluated the public health measures implemented during the organization of an MGE amid the COVID-19 pandemic. The aforementioned analysis considered the Beijing 2022 Games stakeholders from overseas as well as the residents of China.

### Data sources

The daily positive test counts and the type of detection (at the airport or during daily routine testing) were available from the official websites of the International Olympic Committee (IOC) and the Beijing Organizing Committee for the 2022 Olympic and Paralympic Winter Games (BOCOG). Additional data, such as the total number of tests at the airport performed by the China Customs department and the total number of daily screening polymerase chain reaction (PCR) tests performed at authorized laboratories, were extracted from the BOCOG website. As these data were available to the public and were anonymized, ethical approval was not required.

### Study setting

For participants of the Beijing 2022 Games, a comprehensive set of countermeasures to mitigate the risks of COVID-19 transmission were implemented 14 days before their departure from their home country until they left China; these countermeasures comprised the bubble strategy (also called the closed-loop system), pre-departure testing, testing at the airport, daily screening and isolation, the mandatory wearing of N95 masks, and mandatory vaccination and medical documentation (Table 1) (12).

**Table 1:** Primary and supplementary countermeasures implemented in the Beijing 2022 Olympic Winter Games.

**Countermeasures**	**Description**	**Scope of application**
Bubble strategy	A special system with dedicated transport to keep the Games participants and the local citizens apart. Once an individual enters the bubble (or the *closed loop*), all his/her activities will be subject to closed loop system of the same standard covering accommodation, transport, catering, training and competitions, arrival and departure, regardless of his/her identity as foreign nationals or domestic workforce.	Suitable for short-term MGEs or MGEs with heterogeneous disease transmission risks between participants and local residents. Demanding for social resources.
Daily screening test	Daily RT-PCR testing for oropharyngeal swabs. Nasopharyngeal swabs were used for confirmatory test.	Universal. Could be substituted by other test methods as per reality.
Isolation	Once confirmed positive, symptomatic cases would be transferred in the fastest fashion to hospitals and asymptomatic ones to isolation facilities.	Universal. Relies on rapid response and resource reserve.
Mandatory N95 mask	N95 masks wearing without an inhalation valve required all the time except when training/competing, eating, drinking or being alone.	Universal. Suitable for indoor activities. Could be substituted by surgical masks as per risk assessment.
Vaccination	Full vaccination required at least 14 days prior departure. Booster shot encouraged. Exemptions considered for athletes.	Universal.
Pre-departure test	Two COVID-19 (PCR) tests on two separate days within 96 hours of departure.	Universal.
Contact tracing	Flight close contacts and routine contacts in the bubble identified and managed as per the Playbook. Close contacts would live alone, dine alone, transport alone and train alone. Nasopharyngeal tests twice a day.	Universal.
Physical distancing	Two-meter distance from athletes and at least one meter from others required, including in operational spaces.	Universal.
Ventilation	Regularly ventilation performed.	Universal.
Disinfection	Hand sanitizer available at the entrance to all services and spaces. Cleaning and disinfection reinforced in highly populated areas. Preventive disinfection and terminal disinfection performed.	Universal.

### Bubble strategy

An integrated system, involving the Olympic Village, hotels under contract, training venues, competition and non-competition venues, hospitals, banks/automated teller machines (ATMs), and other pre-listed destinations with dedicated linking transport, was established to keep the participants of the Beijing 2022 Games separate from the residents. To maintain the integrity of the bubble, the vehicles shuttling participants of the Beijing 2022 Games between destinations were marked with a specific logo and followed a dedicated route to access other bubbles. Signs and notice boards were placed at the entrance of each bubble, and the security staff checked the accreditation cards of the entrants. Participants of the Games who were permitted access were able to enter and play their role inside the bubble, as well as shuttle from one bubble to another. Individuals without access were denied entry into the bubbles.

### Three-layer testing strategy

The Beijing 2022 Games implemented a three-layer testing strategy to minimize the risk of COVID-19 transmission, including pre-departure tests, testing at the airport, and daily screening tests. The pre-departure tests comprised two COVID-19 PCR tests on two separate days within 96 h of departure. Additional requirements were applied for individuals who were previously infected and for those who were incompletely vaccinated as per the Playbooks jointly developed by the IOC and the BOCOG. The Playbooks served as the basis of the Beijing 2022 Games plan to ensure that all participants acquired an understanding of the principles and specific COVID-19 countermeasures, such as vaccination, closed-loop system, COVID-19 liaison officers, testing and isolation, and health practices throughout the whole journey, comprising the pre-departure period, entry into China, during the Beijing 2022 Games, and until departure from China. The Playbooks were jointly developed by the IOC and the BOCOG in close collaboration with the Chinese government and relevant authorities. Two Playbooks were developed for the Beijing 2022 Games: one for the athletes and team officials, and another for other stakeholders including International Federations, broadcasters and press, marketing partners, Olympic and Paralympic Family members, and international and domestic workforces.

Commercial SARS-CoV-2 RNA RT-PCR testing kits were used to detect the *N* and *ORF1ab* genes, with the *RNP* gene used as the internal reference. The cutoff cycle threshold value for positivity was initially 40 as indicated in the instructions of the assay kits but it was later adjusted to 35 on 23 January 2022 as the Beijing 2022 Games progressed. A positive specimen was defined as a specimen that was positive for both the *N* and *ORF1ab* genes. As regards the test to be conducted on arrival at the airport, all participants entering China to take part in the Beijing 2022 Games had both nasopharyngeal and oropharyngeal swabs taken to achieve a higher PCR test sensitivity, and the samples were sent to the official laboratory of the China Customs department. For testing PCR daily, all participants of the Beijing 2022 Games had either a nasopharyngeal or an oropharyngeal swab taken; if the screening test was positive, an additional nasopharyngeal swab was collected for confirmatory testing.

On 23 January 2022, the Medical Expert Panel for the Beijing 2022 Games (MEP) released an additional interpretation of test results. Among individuals who tested positive for both the *N* and *ORF1ab* genes with a cutoff cycle threshold value of >35, an asymptomatic person would be managed as a close contact and to continue his/her activities, while a symptomatic person would be sent to a dedicated hospital for further diagnostic testing and treatment. Enhanced countermeasures were applied for those who managed as close contacts to minimize the risk of transmission depending on stakeholder types, such as to dine alone, to live in a single room, to use dedicated vehicles to shuttle between locations, and to have COVID-19 (PCR) tests done every 12 hs for seven consecutive days (12).

The nucleic acid testing plan was intended to cover 100% of the Beijing 2022 Games stakeholders. However, due to the complexity of the Beijing 2022 Games, arrangements, and fully scheduled activities, some participants could have missed some tests. To minimize this problem, an automated algorithm with specific testing requirements as per the participant's status (close contact/non-close contact) was integrated into the checkpoint at the entrance of all hotels/venues. Any individual who missed a nucleic acid test the previous day would be reminded to undergo the same test at his/her earliest convenience without discontinuing their role in the Beijing 2022 Games. Furthermore, all hotels were equipped with public health teams that were able to initiate an on-site response. One of the responsibilities of these public health teams was to check all daily test records and issue reminders to any individual with missing test results.

### Wearing of N95 masks or masks of an equivalent standard

N95 masks are proven to be effective against respiratory infectious diseases. In line with the WHO guidelines, all participants of the Beijing 2022 Games were required to constantly wear an N95 mask (KN95, N95, FFP2, or any other mask of an equivalent standard) without an inhalation valve, except when they were training/competing, eating, drinking, or being alone (13). The public health team and volunteers at venues were responsible for reminding anyone who was not wearing a mask or not wearing a mask properly.

### Vaccination requirement

Vaccination has proven to be the ultimate tool for effectively reducing the COVID-19 infection, severity, hospital admission, and transmission. The Beijing 2022 Games required all participants to either receive full vaccination at least 14 days prior to their departure from their home country or, as per China's general entry policy in place at the time, enter a 21-day quarantine period before entering the bubble. In addition, the second version of the Playbooks encouraged individuals to receive a booster dose of the vaccination. The immunization schedule and procedure were in accordance with the requirements of the participant's country/region of residence or the national health authority where the vaccine was administered.

Athletes and team officials with medical contraindications to COVID-19 vaccination were granted exemption from vaccination on a case-by-case basis after evaluation by the MEP. The criteria for exemption from vaccination were clearly indicated in the Playbook. Athletes or team officials seeking exemption from vaccination were required to send an application to the MEP, which would decide on the request and come back within 7 days. The MEP held weekly meetings before the Beijing 2022 Games and also held meetings upon request to deal with urgent matters during the Games.

## Results

**Figure 1:**
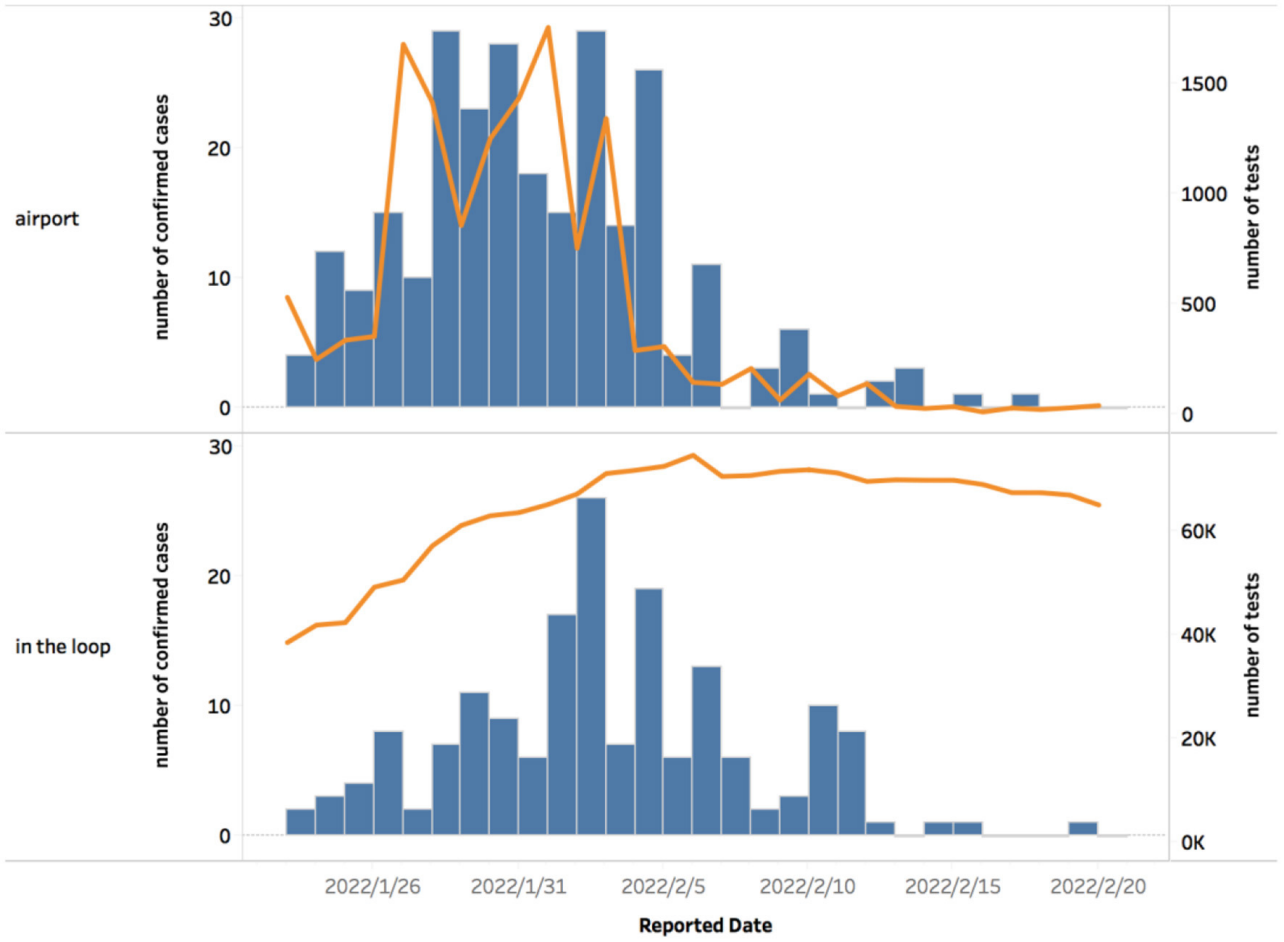
The number of confirmed positive cases and screening tests performed during the Beijing 2022 Olympic Winter Games. This figure depicts the changing patterns in daily reported positive cases and the number of nucleic acid tests performed from 23rd January 2022 to 20th February 2022. The histogram represents the number of daily reported cases while the line graph depicts the number of nucleic acid tests conducted per airport and within the loop. The number of reported cases from the airport went with the numbers of tests conducted, namely the numbers of entry personnel. Since 3rd February 2022, the number of tests conducted in the loop stayed at around 70,000.

A total of 13,690 Beijing 2022 Games stakeholders entered China from 23 January 2022 to 20 February 2022, with a peak of 1,759 persons per day. As per the statistics released by the BOCOG, the number of COVID-19-positive cases grew in tandem with the number of inbound Beijing 2022 Games stakeholders to China, indicating the timeliness of the detection of infection (14). A total of 1,859,423 routine PCR tests were performed in the bubble. From 3 February 2022, the number of tests conducted per day stabilized at around 70,000 (Figure 1).

**Figure 2:**
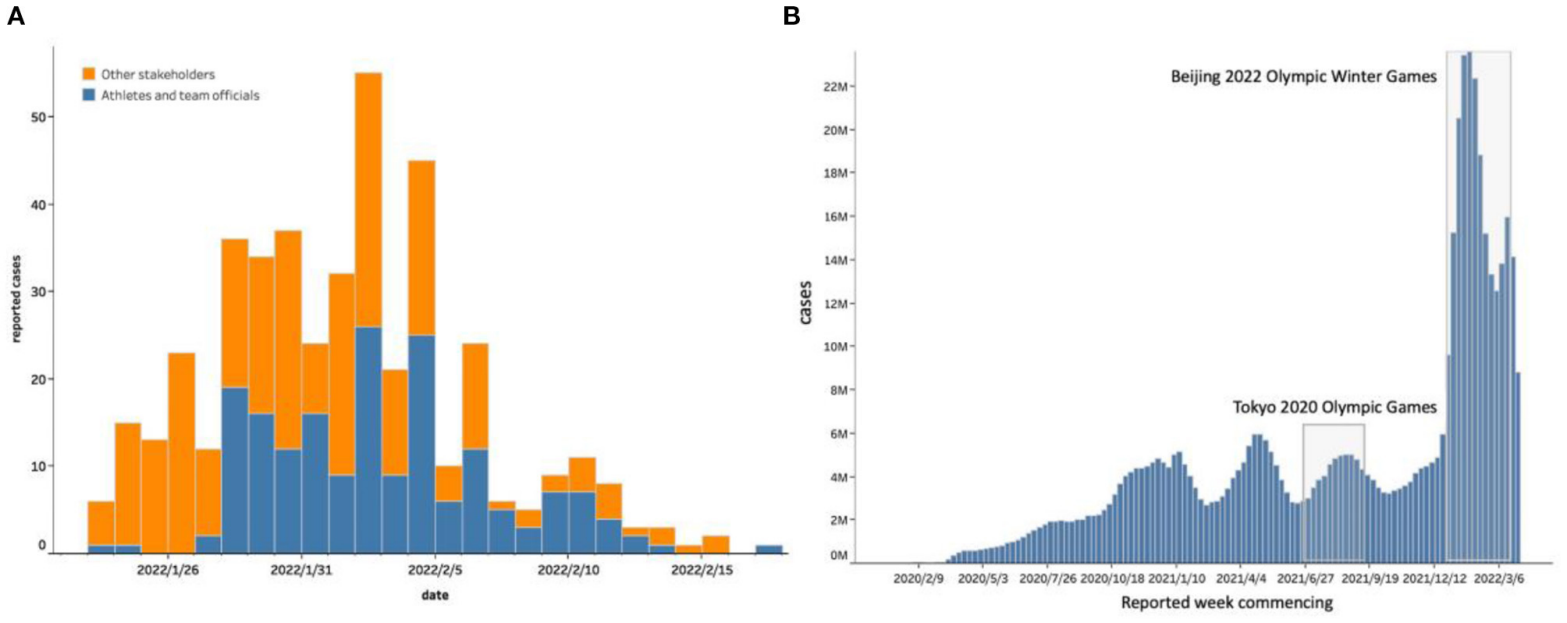
The number of confirmed positive cases by stakeholder types during the Beijing 2022 Olympic Winter Games. **(A)** Depicts the epidemiological curve of coronavirus disease 2019 (COVID-19) cases during the Beijing 2022 Olympic Winter Games, broken down by stakeholder types. **(B)** Shows the context when the Beijing 2022 Olympic Winter Games were held, namely during the highest peak of COVID-19 cases.

In total, 437 COVID-19-positive cases were detected from 23rd January to 20th February 2022 (Figure 2) (15). Outside the bubble, no Beijing 2022 Games stakeholders, such as the spectators, some marketing partners, and a number of workforces, tested positive in regular nucleic acid testing.

### Temporal distribution of cases

There was a single peak of COVID-19-positive cases during the Beijing 2022 Games. The number of positive cases increased in accordance with the number of Beijing 2022 Games stakeholders entering China. By the day of the Opening Ceremony (4th February 2022), 80.8% of the positive cases had already been identified, and no further infections were discovered around the Closing Ceremony (20th February 2022) (14). The majority of cases were detected before the Opening Ceremony, matching stakeholder entry patterns.

### Mode of detecting an infection

During the Beijing 2022 Games, the COVID-19-positivity rate at the airport was 1.9%, which was substantially higher than that of the Tokyo 2020 Olympic Games (0.2%). In addition, sporadic cases were found outside the bubble in Beijing, with 20 being the highest number of daily new infections. Of the positive cases among participants of the Beijing 2022 Games, 60.6% were detected through airport screening and 39.4% were detected through routine screening tests. Nearly 92.0% of the cases were detected within 7 days of arrival, indicating that most of the infections were contracted overseas and that secondary infections were well controlled via comprehensive countermeasures.

### Demographic distribution of cases

From 23 January to 20 February 2022, a total of 437 positive cases were detected inside the bubble, among which 22.4% (98/437) were athletes, 19.9% (87/437) were team officials, and 57.7% (252/437) were other stakeholders. No spectators, marketing partners, or workforces staying outside the bubble tested positive for COVID-19 during this period.

According to a report by the Beijing Municipal Health Commission, four local residents in the bubble became infected with COVID-19. No local cases within the bubble were reported from 7 days after the Closing Ceremony, which exceeded the incubation period of the Omicron variant, indicating the effectiveness of countermeasures implemented during the Beijing 2022 Games.

## Discussion

The Beijing 2022 Games were held in an unprecedented era in which the SARS-CoV-2 Omicron variant was rampant worldwide. This novel coronavirus variant, which may be able to circumvent the defenses built up by vaccination or previous infection, posed a global threat to public health. At this very crucial moment, the Beijing 2022 Games aimed to bring people from different countries *together*. Amid the surge in global COVID-19 infections, the Beijing 2022 Games were carried out as scheduled. Over 70,000 people strictly abided by the COVID-19 policies developed to control the source of infection, block the transmission, and protect susceptible individuals. The success of the Beijing 2022 Games with minimal COVID-19 transmission demonstrated the possibility of holding global MGEs during future pandemics. Our analysis of the COVID-19 cases detected during the Beijing 2022 Games put forth two main findings. First, the source of most of the infections was overseas. The detection of 60.6% of the cases through airport screening and detection of 92.0% of the cases within 7 days after arrival indicated that most of the cases were detected at an early stage, with limited opportunity for viral shedding and transmission. Second, no signs of infection spreading inside the bubble or spreading from within the bubble to the outside were noticed. During the Beijing 2022 Games, the number of infections decreased before the Opening Ceremony and the last case was detected on 18 February 2022, which was 2 days before the Closing Ceremony. These findings suggest that the Beijing 2022 Games successfully achieved a minimal risk of disease transmission amid the COVID-19 pandemic. This achievement was attributable to the bubble strategy, the three-layer PCR testing, the mandatory wearing of N95 masks, and mandatory vaccination.

The bubble strategy was adopted at several events and required stringent enforcement to achieve the full effect (16). In contrast to the Tokyo 2020 Olympic Games where the stakeholders could temporarily exit the bubble and eventually be free to move after 14 days, the participants of the Beijing 2022 Games had to either remain within the bubble or complete the quarantine period before they could exit the bubble to interact with the local residents. According to the statistics released by the IOC and the BOCOG, the maximum number of people was ~75,000 people per day within the bubble. The core essence of the bubble strategy is to maintain its integrity without unsupervised interactions with those outside the bubble. Therefore, the effectiveness of a core strategy relies largely on its stringent implementation and supervision, which places huge demands on social resources and social mobilization ability. As a result, the bubble strategy is most practical when it comes to dealing with short-term MGEs or MGEs with heterogeneous disease transmission risks between the participants and the residents; that is, when the risk level of the entrants is substantially higher or lower than that of the residents.

Traditionally, the three pillars of epidemic control are direct control or elimination of agents at the source of transmission, breaking the transmission chain and protecting the susceptible population. The three-layer testing strategy was extremely important for minimizing the source of infection in the Beijing 2022 Games bubble by reducing the risk of infection. Mass screening testing has proven to be an effective approach for controlling the COVID-19 pandemic due to its excellent ability to detect pre-symptomatic and asymptomatic cases (17). However, the collection of oropharyngeal and nasopharyngeal samples might not be pragmatic for some countries/organizations or scenarios; therefore, other types of tests, such as antigen tests, may be a reasonable option. Modeling research suggests that effective screening depends largely on the testing frequency and the turnaround time, and is only marginally improved by a higher test sensitivity (18). Therefore, organizations and governments might have more choices of testing methods when holding MGEs.

Wearing N95 masks or masks of an equivalent standard was mandatory during the Beijing 2022 Games to break the transmission chain. To our knowledge, this is the first real-world high-profile sporting event enforcing such a mask-wearing requirement. Previous sporting events required the use of cloth masks or surgical masks (19). A randomized controlled study conducted in Spain to evaluate the efficacy of continuous N95 mask-wearing and same-day SARS-CoV-2 antigen test screening at a live indoor concert with over 500 people in attendance found no positive COVID-19 cases within 8 days after the event in the intervention group in which physical distancing was not required and singing and dancing were permitted (20). Guidelines and meta-analyses recommend the use of N95 masks in high-risk settings/populations, such as for those medical staff caring for patients with COVID-19, and suggest that significant differences were found in the effectiveness of surgical masks vs. N95 masks among the general public (21–23). However, most of the studies were conducted in the community or hospital settings, while none of them were conducted at MGEs (22–24). Although the three-layer testing strategy lowered the infection risk gradually, there was a chance that infections could not be detected in time. The Omicron variant may have rapidly spread within the bubble if additional personal hygienic practices were not adhered to. Every participant had an essential role in the Games and could not afford to become infected and unable to function, especially the athletes and their teams who had prepared for the Beijing 2022 Games for years. Therefore, the Beijing 2022 Games required all participants to wear N95 masks without an inhalation valve. Future MGEs that do not involve high-profile competitions may allow the participants to wear masks of other types, such as surgical masks, and their effects should be analyzed.

Vaccination is the key public health tool against COVID-19 that gives the potential for enhanced protection and reduces the occurrence of a severe clinical condition caused by the SARS-CoV-2 infection (25–27). Among those who completed the full vaccination course, the COVID-19-positive individuals resumed their normal function after a short stay in the dedicated hospitals and isolation facilities, thus lowering the pressure on the medical services in the closed-loop system and minimizing the impact of COVID-19 infection on the Beijing 2022 Games.

Although prophylactic measures safeguarding public health implemented during the Beijing 2022 Games proved to be useful, it is difficult to assess the cost-effectiveness of each countermeasure. As per the balanced budget published by the Tokyo 2020 Organizing Committee of the Olympic and Paralympic Games, JPY ¥35.3 billion (USD $0.3 billion) were used to implement COVID-19 countermeasures (28). The Beijing 2022 Games had incurred much higher expenses than the Tokyo 2020 Olympic Games owing to the maintenance of the closed-loop system, frequent PCR testing, use of N95 masks, and provision of dedicated hospitals and isolation facilities. These expenses are a great challenge to most countries. Nevertheless, the Beijing 2022 Games provided an irreplaceable experience in organizing such a successful international MGE by implementing a set of public health countermeasures to contain the transmission of SARS-CoV-2 during the MGE and maintain the public health security of the host country. This experience provides a valuable public health legacy for future events during an emerging infectious disease pandemic.

## Data availability statement

The raw data supporting the conclusions of this article will be made available by the authors, without undue reservation.

## Author contributions

Conception and design: YS and DH. Analysis and interpretation of the data: TY, TZ, YS, and DH. Drafting of the article: RL, YT, TG, TY, and TZ. Critical revision of the article for important intellectual content: QW, YS, and DH. Collection and assembly of data: DH, TZ, and YT. All authors contributed to the article and approved the submitted version.
